# High-speed scanning of planar images showing ^123^I-MIBG uptake using a whole-body CZT camera: a phantom and clinical study

**DOI:** 10.1186/s13550-019-0491-z

**Published:** 2019-02-26

**Authors:** Nanami Okano, Iichiro Osawa, Saki Tsuchihashi, Masafumi Takahashi, Mamoru Niitsu, Ichiro Matsunari

**Affiliations:** 1 Department of Radiology, Saitama Medical University Hospital, 38 Morohongo, Moroyama, Iruma, Saitama, 350-0495 Japan; 2 Department of Central Radiological Technology, Saitama Medical University Hospital, Saitama, Japan; 3 Division of Nuclear Medicine, Department of Radiology, Saitama Medical University Hospital, Saitama, Japan

**Keywords:** CZT, HMR, MIBG, Planar images, LBD

## Abstract

**Background:**

The heart-to-mediastinum ratio (HMR) obtained in myocardial sympathetic innervation imaging using ^123^I-metaiodobenzylguanidine (MIBG) is used for heart failure or Lewy body diseases (LBD). Discovery NM/CT 670 CZT, a novel whole-body scanner, enables direct HMR measurements in planar images, in contrast to cardiac-dedicated CZT-based cameras which require specific post-processing reconstruction. We sought to investigate the clinical utility of the Discovery NM/CT 670 CZT for myocardial innervation imaging and the potential time reduction.

**Results:**

Following preliminary phantom examinations, ^123^I-MIBG planar imaging was performed in 36 patients with suspected or known LBD to measure HMRs with a collection time of 300 s. Images for different collection times were subsequently reframed using already acquired data, and changes in HMRs were evaluated.

The HMRs for patients with versus without clinically diagnosed LBD were 1.63 ± 0.08 versus 2.21 ± 0.08 at early phase (*p* < 0.001) and 1.54 ± 0.09 versus 2.08 ± 0.09 at delayed phase (*p* < 0.001). The difference of HMRs (300 s − other collection time) became greater as the collection time became shorter. There was good consistency in HMRs between the 300-s images (reference) and the 200-s (intra-class correlation (ICC) coefficients > 0.99), 100-s (ICC coefficients > 0.97), and 50-s (ICC coefficients > 0.89) images.

**Conclusions:**

In planar images with a whole-body CZT-based camera, the HMRs of patients with LBD were significantly lower than those without. HMRs with the collection time of 50 s and longer showed good consistency with those of 300 s in the ICC analysis. These findings indicate a clinical utility of this novel scanner for HMR measurements and potential time reductions.

**Electronic supplementary material:**

The online version of this article (10.1186/s13550-019-0491-z) contains supplementary material, which is available to authorized users.

## Background

Myocardial sympathetic innervation imaging using ^123^I-metaiodobenzylguanidine (MIBG) has been used to predict long-term outcomes in patients with heart failure [1, 2] and, recently, to diagnose Lewy body diseases (LBD) such as Parkinson’s disease (PD) and dementia with Lewy bodies (DLB) [3]. The heart-to-mediastinum ratio (HMR) evaluated by myocardial sympathetic imaging using the Anger camera can stratify the mortality risk of heart failure [1] and reflect the clinical severity of LBD [4, 5]. Meanwhile, the cadmium-zinc-telluride (CZT) camera, which was recently introduced for use in clinical settings, has demonstrated greater sensitivity (partially attributable to its cardiac-centred design), higher energy resolution, shorter acquisition time, and/or less radiation exposure compared with the Anger camera [6–9]. Studies have reported evidence for HMR values calculated from myocardial innervation imaging using cardiac-dedicated CZT-based cameras such as D-SPECT (Spectrum Dynamics, Israel) and Discovery 530 C (prototype of Discovery family) [10, 11]. As HMR measurements are based on planar images of the thorax [10, 12], these cardiac-dedicated CZT-based cameras that lack planar images require special reconstruction techniques for HMR calculations [10, 11].

In contrast, a novel CZT camera, Discovery NM/CT 670 CZT SPECT (GE Healthcare, Milwaukee, Wisconsin), that was designed for whole-body examination, has become commercially available and enables direct calculation of the HMR in planar images. Therefore, by skipping multiple post-processing reconstructions, the Discovery NM/CT 670 CZT single-photon emission computed tomography (SPECT) may be theoretically preferable over cameras with cardiac-dedicated detectors for objective quantification of HMR. However, application of the whole-body CZT camera for myocardial innervation imaging has not been studied yet.

Moreover, some studies have also provided evidence of several-fold time reduction with the CZT-based scanners in comparison with Anger cameras, mainly in myocardial perfusion imaging [13, 14], which would reduce the time for patients to remain motionless or reduce the radiation exposure. However, studies on time reduction for myocardial innervation imaging have not been published yet. The potential time reduction can be evaluated by comparing the images and quantitative values obtained at various collection times, and this processing can be readily performed after examinations using the Lister tool (GE Healthcare); the Lister tool allows post hoc reframing of already acquired data without additional radioactive tracer administration or imaging acquisition.

In the current phantom and clinical study, we sought to demonstrate (1) the utility of the Discovery NM/CT 670 CZT, a whole-body scanner, for myocardial innervation imaging, and (2) the potential time reduction achieved by comparing images and HMRs at different collection time settings

## Methods

### Phantom models

A planar MIBG phantom (custom-made device, Taisei Medical Co., Ltd., Osaka, Japan; Hokuriku Yuuki Co., Ltd., Kanazawa, Japan) for normal and disease models, which was composed of acrylic plates and parts representing organs such as the heart, mediastinum, lung, and liver, was used (Additional file 1: Figure S1A). As all organ parts were connected as one compartment, no adjustment of radionuclide concentration for each organ part was required. This phantom has been used in several institutions, and we prepared it in the same manner as in previous studies [15, 16]. The thickness of the vacant space at heart/mediastinum was 30/10 and 25/15 mm in the normal and disease models, respectively [16] (Additional file 1: Figure S1 B and C).

### Subjects in clinical study

Data from 36 consecutive patients with suspected or known PD or DLB who underwent MIBG myocardial innervation imaging using the Discovery NM/CT 670 CZT between July 25 and November 31, 2017, were retrospectively reviewed.

### MIBG myocardial innervation imaging and analysis protocol

Using the Discovery NM/CT 670 CZT camera, anterior and posterior planar imaging was performed after injection of 55 MBq of ^123^I-MIBG for the phantom model. In the clinical study, anterior planar imaging was performed 15 min (early) and 3 h (delayed) after intravenous injection of 111 MBq of ^123^I-MIBG. The Lister tool on the Xeleris 4.0 workstation (GE Healthcare) allowed post hoc reframing of already acquired data with various parameters such as energy window widths and collection times, without additional radioactive tracer administration or imaging acquisition. All images were collected with matrices of 512 × 512 and at an energy peak of 159 keV.

**Fig. 1:**
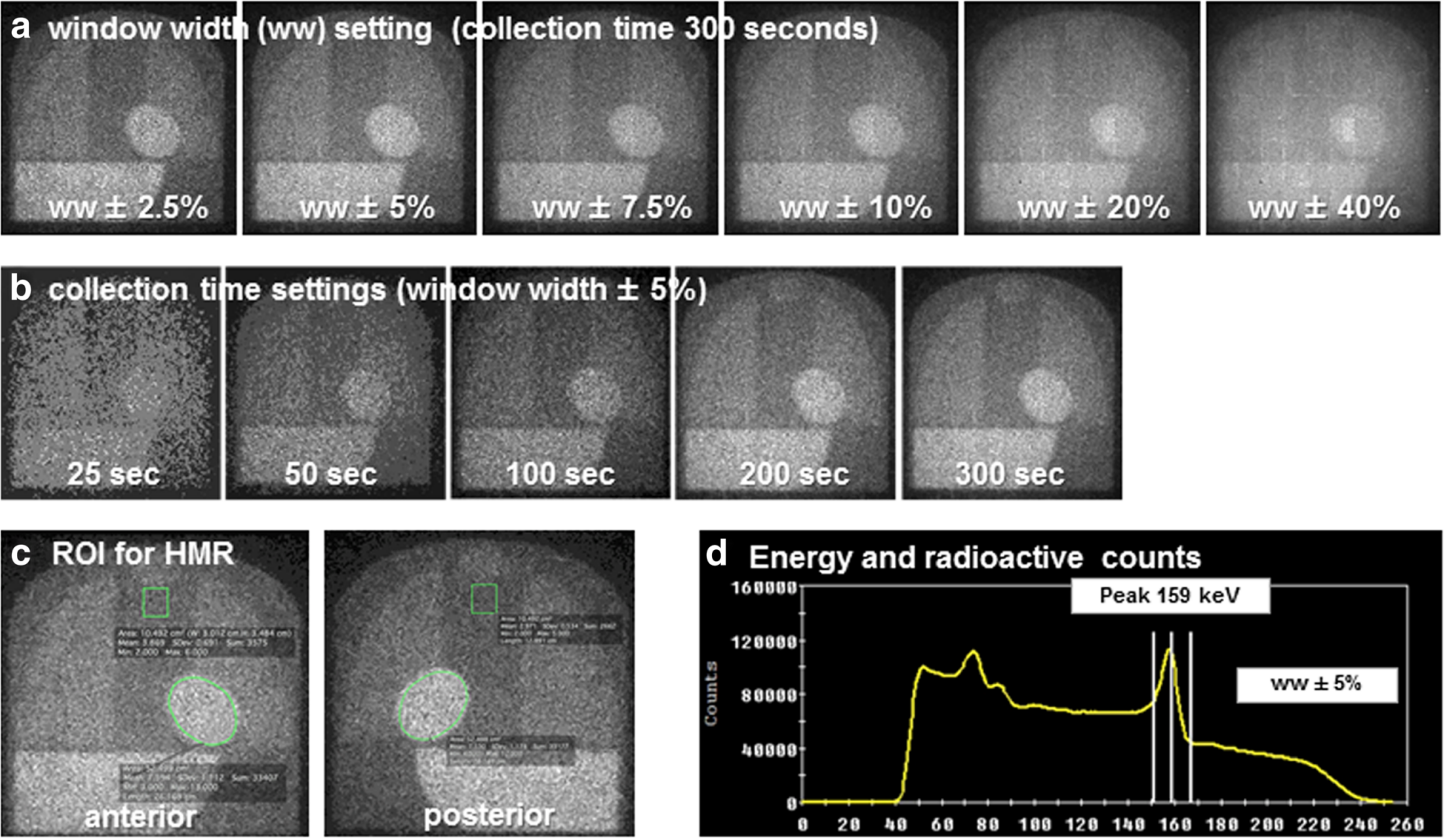
Image processing and HMR calculation. **a** Images with different energy window widths (symmetrical, ± 2.5%, 5.0%, 7.5%, 10%, 20%, and 40%) using a fixed collection time (300 s). **b** Images with different collection times (25, 50, 100, and 200 s) using a fixed window width (± 5.0%). **c** The regions of interest (ROIs) manually set on the heart (52.5 cm^2^, oval-shaped) and mediastinum (10.5 cm^2^, square-shaped) in the anterior and posterior planar views. **d** Relationship between energy (*x*-axis) and radioactive counts (*y*-axis) and set energy peak ± window widths (vertical white lines)

In phantom studies, reference images with a collection time of 300 s were acquired, and these images were reframed with different energy window widths (symmetrical, ± 2.5%, 5.0%, 7.5%, 10%, 20%, and 40%) (Fig. 1a). Subsequently, images with a fixed window width (± 5.0%) were reframed with different collection times (25, 50, 100, and 200 s) (Fig. 1b). The regions of interest (ROIs) were set manually on the heart (52.5 cm^2^, oval-shaped) and mediastinum (10.5 cm^2^, square-shaped) in the anterior and posterior planar views, and the HMR values were calculated using the averaged radioactive counts in these ROIs (Fig. 1c) [17]. A schema of the settings for energy peak and window width and radioactive counts is shown in Fig. 1d. To compare properties between the CZT-based and Anger cameras, images with a fixed window width (± 7.5%) were obtained with different collection times (25, 50, 100, 200, and 300 s) using Picker Prism 2000 equipped with low-energy ultra-high-resolution (LEUHR) and medium-energy general-all-purpose (MEGAP) collimators.

In clinical studies, using a fixed energy peak and window width (159 keV ± 5%), images with the reference collection time of 300 s were first acquired and images with different collection times (25, 50, 100, and 200 s) were subsequently reframed. The HMR was calculated in the same way as in the phantom studies but using only anterior views.

### Statistical analysis

Continuous and discrete variables are reported as mean ± standard deviation (SD). Categorical variables are reported as percentage (%) values. Normality was evaluated using the Shapiro-Wilk test. Continuous values were compared between groups using analysis of covariance. In phantom studies, coefficient of variation (CV) values was calculated using the average and SD counts for ROIs at the heart and mediastinum to evaluate the appropriate energy window width (Fig. 1c). In clinical studies, using intra-class correlation (ICC; two-way mixed-effects model, for consistency) coefficient statistics and Bland-Altman analysis with limit of agreement, the correlation and bias between HMRs in images with the reference collection time of 300 s and those with other collection times (25, 50, 100, and 200 s) were evaluated. Statistical analyses were performed using JMP Pro 11.2.0 (SAS Institute Inc., Cary, NC, USA) or SPSS statistics software (version 25, IBM Corp., Armonk, NY, USA), as appropriate.

## Results

### Phantom studies

#### Influence of window widths on the radioactive counts and HMR

**Fig. 2:**
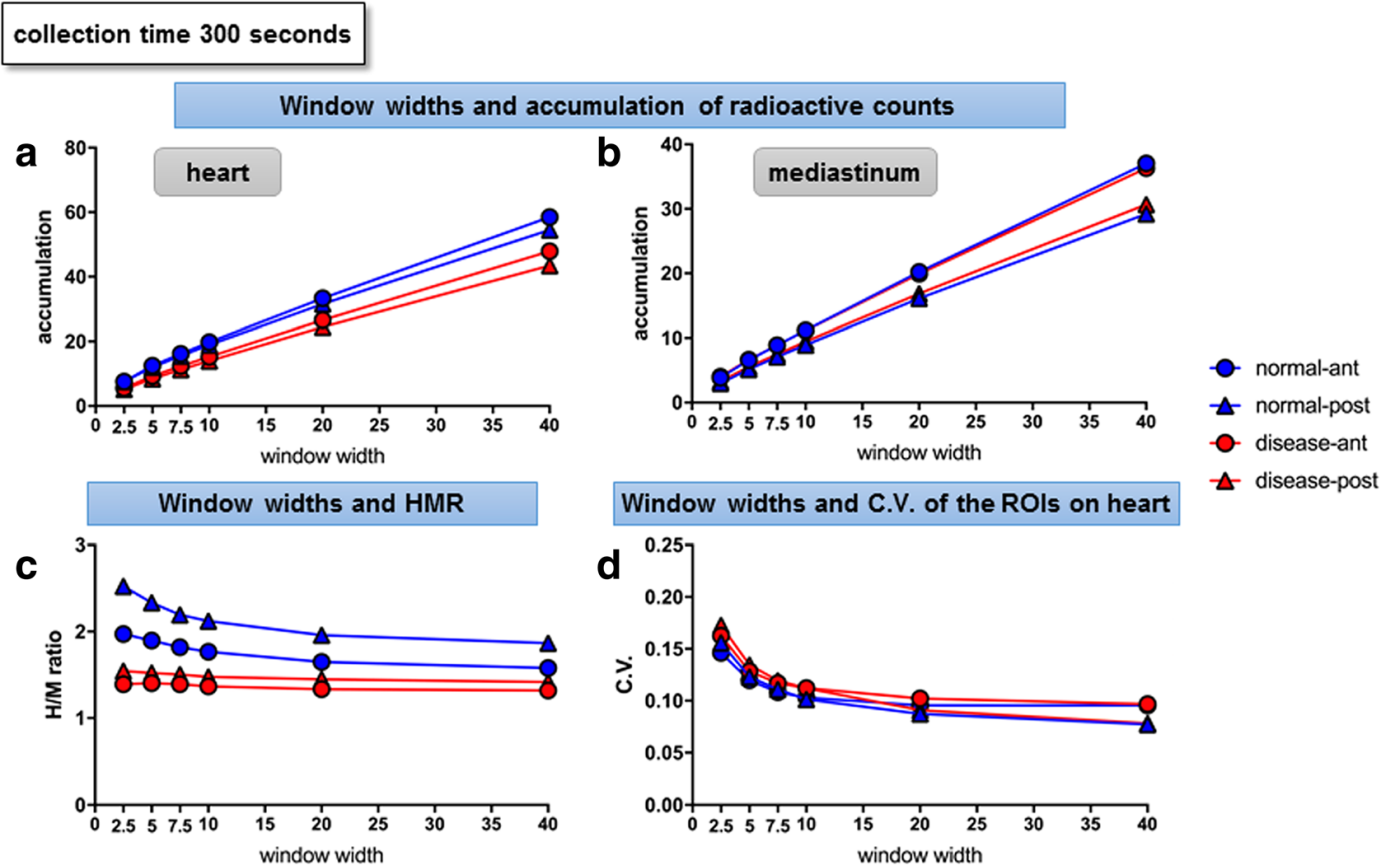
Changes in parameters at different window widths (phantom study). With the collection time fixed at 300 s, the relationships between different window widths (*x*-axis) and radioactive counts in the ROIs on the heart (**a**) and mediastinum (**b**), HMR (**c**), and CV of the ROIs on the heart (**d**), are shown. Blue circles—normal model, anterior view; blue triangles—normal model, posterior view; red circle—disease model, anterior view; red triangle—disease model, posterior view

The MIBG radioactive counts in the ROIs on the heart and mediastinum in anterior and posterior views showed a positive proportional relationship with the window widths in the normal and disease phantom models (Fig. 2a, b).

The HMR decreased as the window widths increased in the normal model, whereas they remained low in the disease model. Thus, the difference in HMR between the normal and disease models was the largest when the window width was set at the smallest (± 2.5%), and the difference reduced as the window widths increased (Fig. 2c). The CV of the radioactive counts for ROIs on the heart also reduced as the window widths increased; the slope of the CV was steep in phases with smaller window widths, in contrast with the gradual slope with window widths of ± 5% or larger (Fig. 2d).

The anterior HMR at 300 s using the window width of ± 5% was 1.89 in the normal model and 1.40 in the disease model.

#### Influence of collection times on radioactive counts and HMRs

**Fig. 3:**
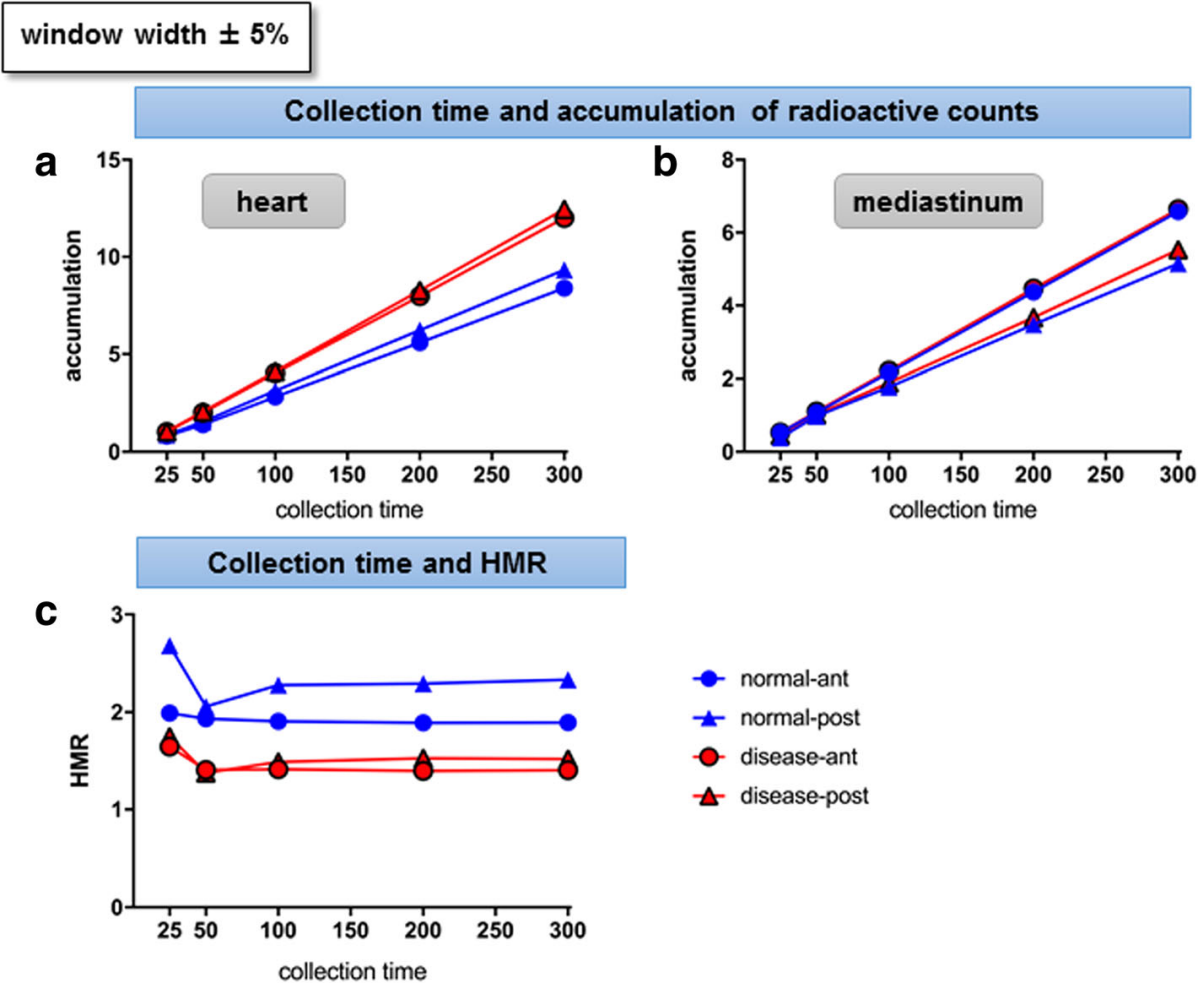
Changes in parameters at different collection times (phantom study). With the window width fixed at ± 5%, the relationship between different collection times (*x*-axis) and radioactive counts in the ROIs on the heart (**a**) and mediastinum (**b**), HMR (**c**), are shown. The meanings of the markers are the same as indicated in Fig. 2

With a window width of 159 keV ± 5%, the accumulation of MIBG counts on both the heart and mediastinum showed a positive proportional relationship with collection time (Fig. 3a, b). The collection time-dependent changes in HMRs diminished as collection time approximated to 300 s; the changes were small when the collection time was 50 s and longer, with the percent change in comparison with the neighbouring HMR ranging from 0.2 to 10.6% (Fig. 3c).

#### Comparison to the conventional Anger cameras

**Fig. 4:**
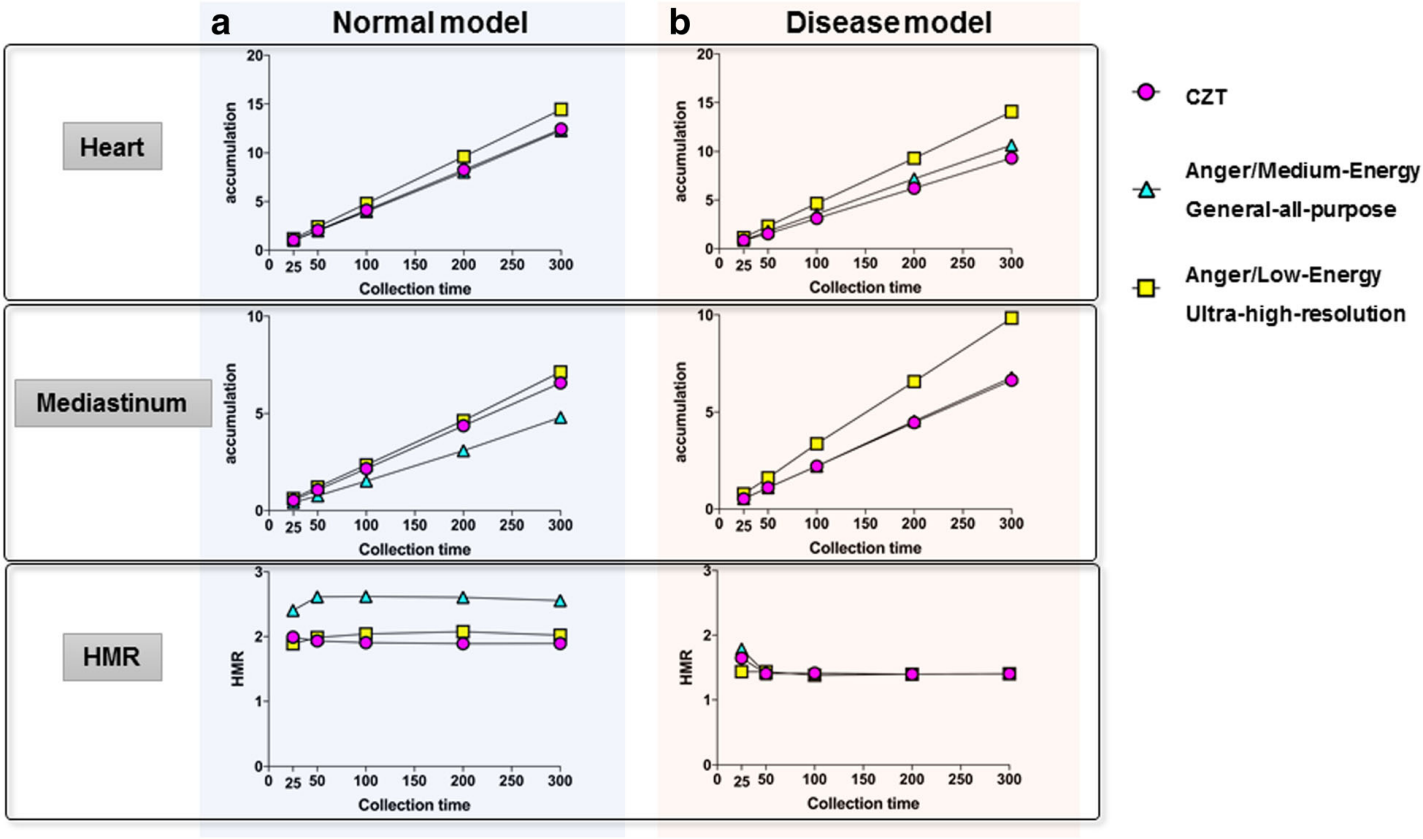
Comparison between CZT-based and Anger cameras (phantom study). The relationship between different collection times (*x*-axis) and radioactive counts in the ROIs on the heart (upper graphs) and mediastinum (middle graphs), and HMR (lower graphs) in the normal (**a**) and disease (**b**) models using three types of cameras. Pink circle—CZT-based camera, light blue triangle—Anger cameras with MEGAP collimators, yellow square—Anger cameras with LEUHR collimators

In the normal model (Fig. 4a), the HMRs obtained from the CZT-based camera were lower than those obtained from the Anger camera with MEGAP collimators, but were comparable to those obtained from the Anger camera with LEUHR collimators. On the other hand, in the disease model (Fig. 4b), the HMRs obtained from these three types of cameras were almost equivalent.

### Clinical studies

#### Characteristics of patients and HMRs

Images of 36 consecutive patients were analysed without exclusion. Patient age was 72.9 ± 8.3 years, with males accounting for 66.7% (*n* = 24). Among these 36 patients, 16 (44.4%) had been diagnosed with PD, 15 (41.7%) with parkinsonism due to other aetiologies, four (11.1%) with dementia due to other aetiologies, and one (2.8%) with DLB. The mean HMRs in the early and delayed phases with a collection time of 300 s using an energy peak ± window width of 159 keV ± 5% were 1.94 ± 0.44 and 1.83 ± 0.46, respectively. The HMRs in patients with clinical LBD (i.e. PD and DLB) were significantly lower than those in patients with other neurological or psychological disease; early HMRs for patients with and without diagnosed LBD were 1.63 ± 0.08 and 2.21 ± 0.08 (*p* < 0.001), respectively, and delayed HMRs for those with and without diagnosed LBD were 1.54 ± 0.09 and 2.08 ± 0.09, respectively (*p* < 0.001).

#### Concordance of HMRs obtained at different collection times

**Fig. 5:**
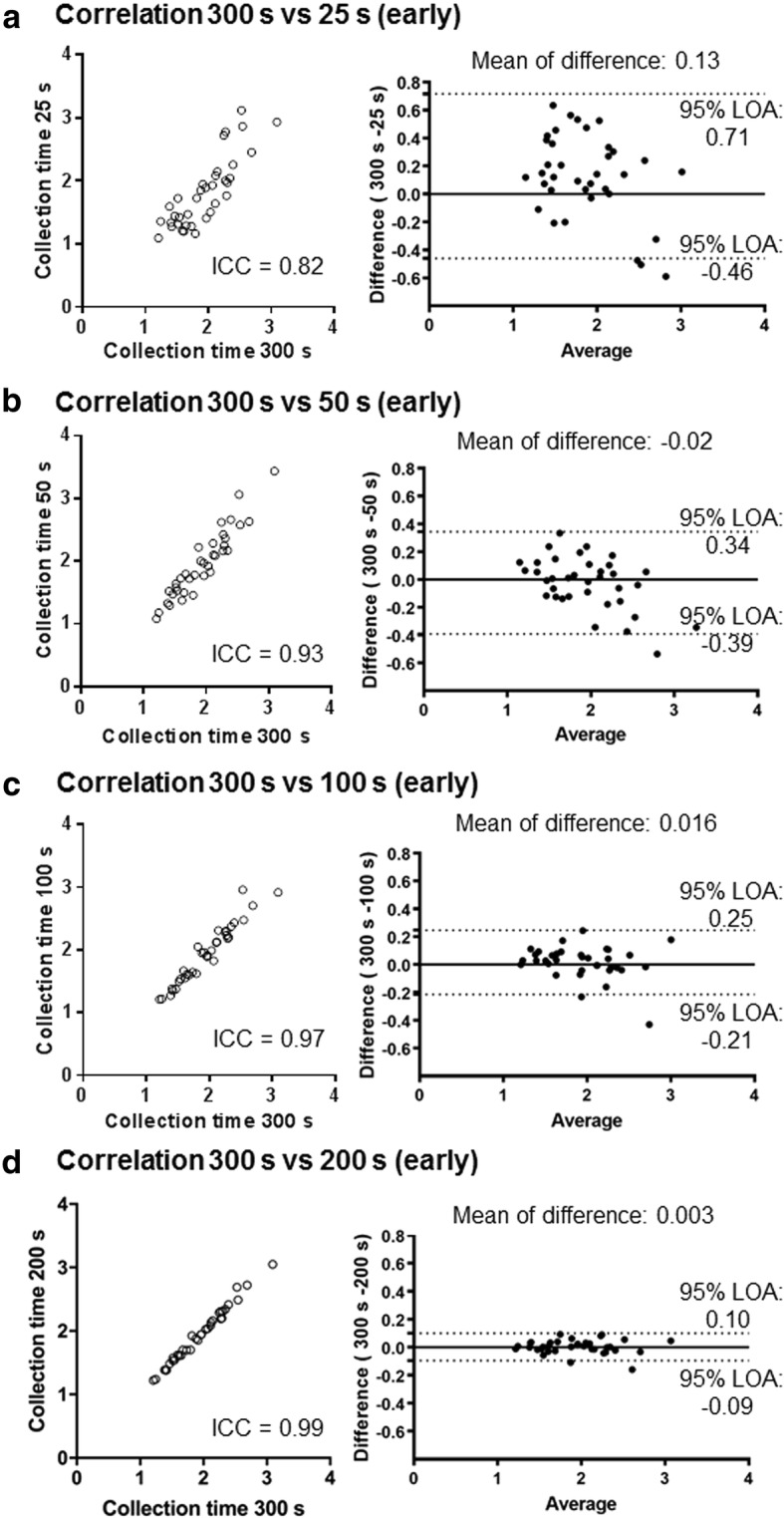
Concordance of early HMRs between different collection times (clinical study). Correlations between the HMRs at early phase with collection times of 300 s (*x*-axis) and 25 (**a**), 50 (**b**), 100 (**c**), and 200 s (**d**) are demonstrated. Bland-Altman analyses show the relationship of the mean (*x*-axis) and difference (*y*-axis) of two corresponding values. ICC intra-class correlation, LOA limit of agreement

**Fig. 6:**
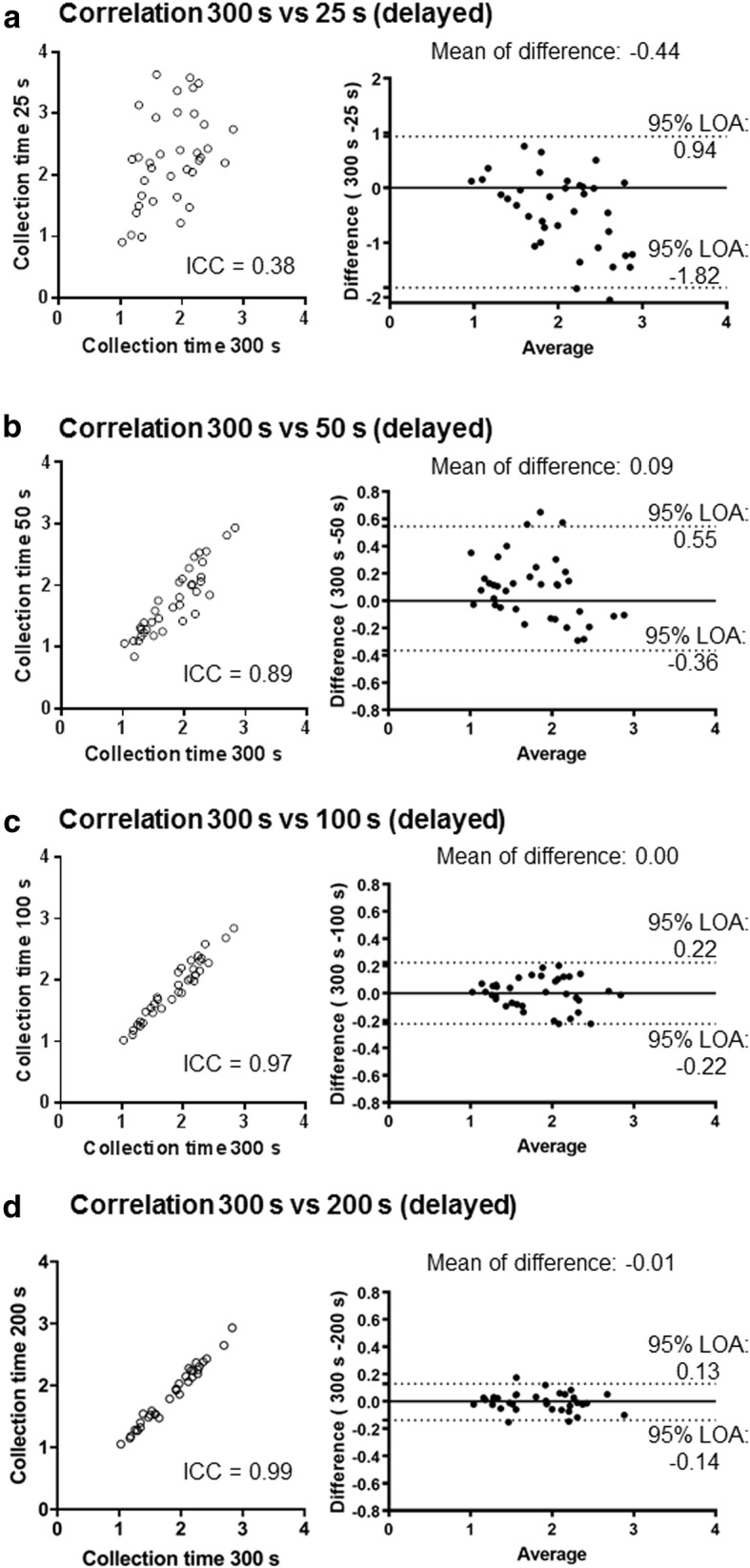
**a**–**d** Concordance of delayed HMRs between different collection times (clinical study). Correlations and Bland-Altman analyses on the HMRs at the delayed phase are shown. See details in the legends of Fig. 5

The ICC (95% confidence interval) values between early HMRs for 300 s and those for 25, 50, 100, and 200 s were 0.82 (0.68–0.90), 0.93 (0.86–0.96), 0.97 (0.94–0.98), and 0.99 (0.99–1.00), respectively (Fig. 5). In the delayed phase, the ICC coefficients (95% confidence interval) between the HMRs for 300 s and those for 25, 50, 100, and 200 s were 0.38 (0.06–0.63), 0.89 (0.80–0.94), 0.97 (0.94–0.99), and 0.99 (0.98–1.00), respectively (Fig. 6). Bland-Altman analysis of the HMRs with a collection time of 300 s and other times showed no obvious time-dependent bias of the differences.

## Discussion

This phantom and clinical study first applied a whole-body CZT camera for myocardial innervation imaging. The clinical study demonstrated the influence of collection time on the HMRs for MIBG myocardial innervation imaging using the Discovery NM/CT 670 CZT, based on the preliminary findings of the phantom study. In patients with known or suspected DLB, the difference of HMRs (300 s − other collection time) became greater as the collection time became shorter. HMRs with a collection time of 50 s and longer showed good consistency with those of 300 s in the ICC analysis, indicating the potential time reduction using this novel scanner.

### Utility of CZT-based detectors for MIBG myocardial innervation imaging

Myocardial sympathetic imaging using the conventional Anger camera has been performed in patients with heart failure [1] or LBD [4, 5]. Recent studies have demonstrated the use of the cardiac-dedicated CZT-based cameras D-SPECT and Discovery 530 C for myocardial innervation imaging [10, 11]. In our phantom study, there was an apparent contrast between HMRs in the disease models and the normal models when the energy window was set narrow at ± 2.5%, although this setting implied heterogeneity of the radioactive counts within ROIs, as represented by the high CV values (Fig. 2). This ‘trade-off’ phenomenon was relieved with energy window widths of ± 5%, where the disease-to-normal contrast was still prominent (0.49 in the anterior views) while the CV reduced (0.13 for the disease and 0.12 for the normal model in the anterior views). These preliminary findings led us to set the energy window width at ± 5% in the clinical study, although previous studies on cardiac-dedicated CZT scanners suggested beneficial effects of asymmetrical window settings [10, 11].

In fact, our clinical study demonstrated significantly lower HMRs in patients with clinically diagnosed LBD in comparison with those in patients with other neurologic diseases, suggesting the feasibility of this new modality for differentiating LBD using HMRs.

The currently commercially available cardiac-dedicated CZT-based cameras, D-SPECT and Discovery NM 530 C, have been known to shorten the collection time by a factor of 3–5 in myocardial perfusion imaging using ^99m^Tc- or ^201^Tl-tracers in comparison with Anger cameras [13, 14, 18]. However, similar evidence of time reduction in myocardial innervation imaging is lacking. Especially, findings showing time reduction using the planar images of the Discovery NM/CT 670 CZT have not been published, except for those involving bone scanning [19]. The reported acquisition time for myocardial innervation imaging with ^123^I-MIBG using these cardiac-dedicated CZT detectors was approximately 10 min [20, 21], similar to that required by Anger cameras [11, 21, 22]. Our study demonstrates that judging from the ICC analysis, HMRs obtained from images with a collection time of 50 s and longer showed good consistency with those obtained with the default 300 s. Meanwhile, as the collection times became shorter, the difference from 300 s became greater along with wider range of 95% limits of agreement in the Bland-Altman analysis. Additionally, we performed further analysis (Additional file 1: Figure S2) that investigated correlation between the HMRs (reference) and the difference of HMRs (300 s − other collection time). As a result, in the early phase collected with 50 s, there was a significant correlation (*r* = − 0.35, *p* = 0.035) between the reference HMRs and the difference of HMRs (300 − 50 s). Thus, disease-to-normal contrast of HMRs might have been preserved with the collection time of 50 s; however, because there were considerable discrepancies of HMRs between 300 and 50 s, such a time reduction cannot be clinically acceptable at this stage. Further studies including regression analysis in larger cohort are needed to elucidate clinical feasibility of such a short collection time. This time reduction for myocardial innervation imaging may be beneficial, because completing the imaging examination may be occasionally challenging for patients as they may suffer heart failure, tremor, or dementia. Additionally, the potential time reduction may also allow administration of lower isotope doses [6–9, 23].

### Comparison with HMRs obtained using previous cameras

In our clinical study, the HMRs of the patients without diagnosed LBD were lower (early phase 2.21, delayed phase 2.08) than those in a previous study using an Anger camera; the HMRs in the early and delayed phases obtained from subjects with low likelihood of cardiac disease (i.e. normal subjects) were 2.39 and 2.49 for low-energy collimators and 2.76 and 3.01 for medium- or low-medium-energy collimators, respectively [24]. Discrepancy between the values obtained from Anger cameras and D-SPECT was also considered; thus, a correction method for standardisation of HMRs has already been reported based on phantom experiments [21]. Thus, HMR may vary depending on the type of collimators. In the Discovery NM/CT 670 CZT, the wide-energy high-resolution (WEHR) collimator was used, in which septal penetration generated from high-energy photons (529 keV) may scatter on mediastinal area, resulting in reduction in the HMR, as observed in the low-energy collimators. Our preliminary phantom study (Fig. 4) supports this speculation; the HMRs obtained from CZT-based camera were lower than those from the Anger camera with MEGAP collimators in the normal model. These results indicate the requirement for future prospective, multi-centre studies for standardisation of the HMRs measured by CZT-based detectors equipped with WEHR for whole-body examination.

### Limitations

This was a single-centre, observational study with a relatively small number of patients. However, we suggest that the evidence provided by our data was relevant enough to warrant further larger studies on the time reduction in myocardial innervation imaging and the standardisation of HMRs when using the novel CZT detectors for whole-body imaging.

## Conclusion

Using Discovery NM/CT 670 CZT SPECT for myocardial innervation imaging of ^123^I-MIBG uptake revealed that the HMRs of patients with LBD were significantly lower than those of patients without LBD. Further, HMRs with a collection time of 50 s and longer showed a good consistency with those with a collection time of 300 s in the ICC analysis, indicating potential time reductions using this novel scanner.

## Additional file

Additional file 1: **Figure S1**. Details of phantom models. **Figure S2**. Correlation between reference HMRs and difference of HMRs (300 s − other collection time) (PDF 407 kb)

## Abbreviations

CV: Coefficient of variation
CZT: Cadmium-zinc-telluride
DLB: Dementia with Lewy bodies
HMR: Heart-to-mediastinum ratio
ICC: Intra-class correlation
LBD: Lewy body diseases
LEUHR: Low-energy ultra-high resolution
MEGAP: Medium-energy general-all-purpose
MIBG: ^123^I-metaiodobenzylguanidine
PD: Parkinson’s disease
SD: Standard deviation
SPECT: Single-photon emission computed tomography
