# Supplementary material for: High-speed scanning of planar images showing 123I-MIBG uptake using a whole-body CZT camera: a phantom and clinical study
Source: EJNMMI Res. 2019 Feb 26;9:22. doi: 10.1186/s13550-019-0491-z (PMC6391509; doi:10.1186/s13550-019-0491-z)

Figure S1: Details of Phantom models

A

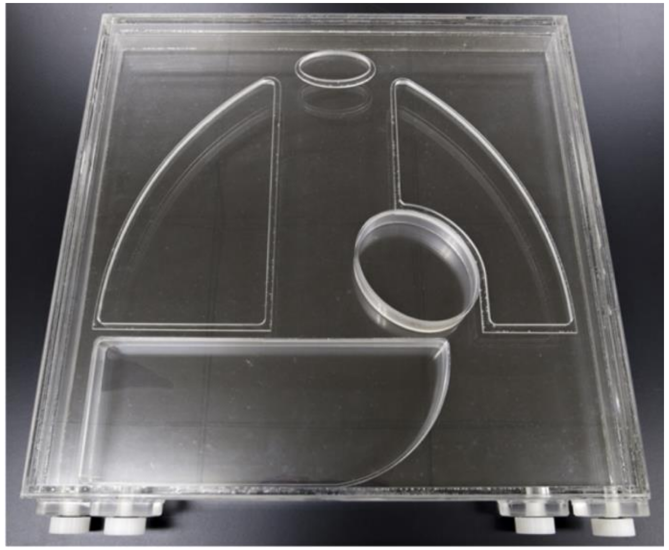

B: Schema of organ model

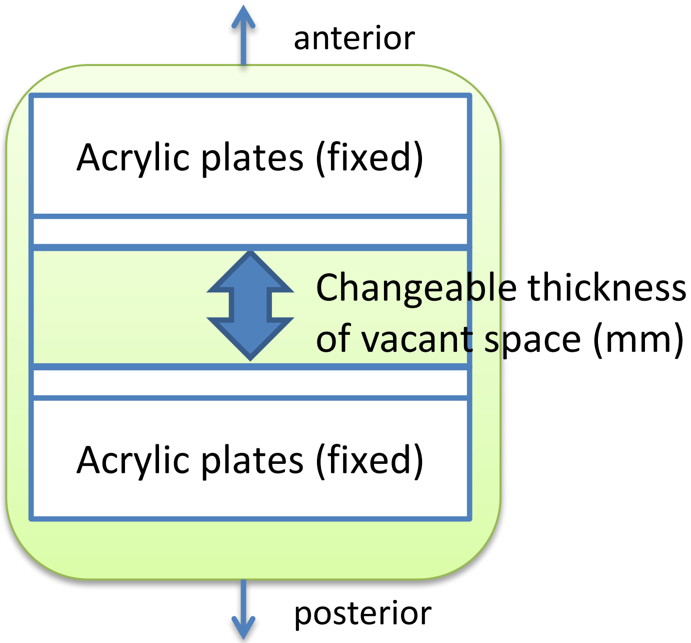

C

Model type	Vacant space thickness (mm)			
	heart	mediastinum	Lung	Liver
Normal model	30	10	15	30
Disease model	25	15	20	30

Figure S2: Correlation between reference HMRs and difference of HMRs (300s – other collection time)

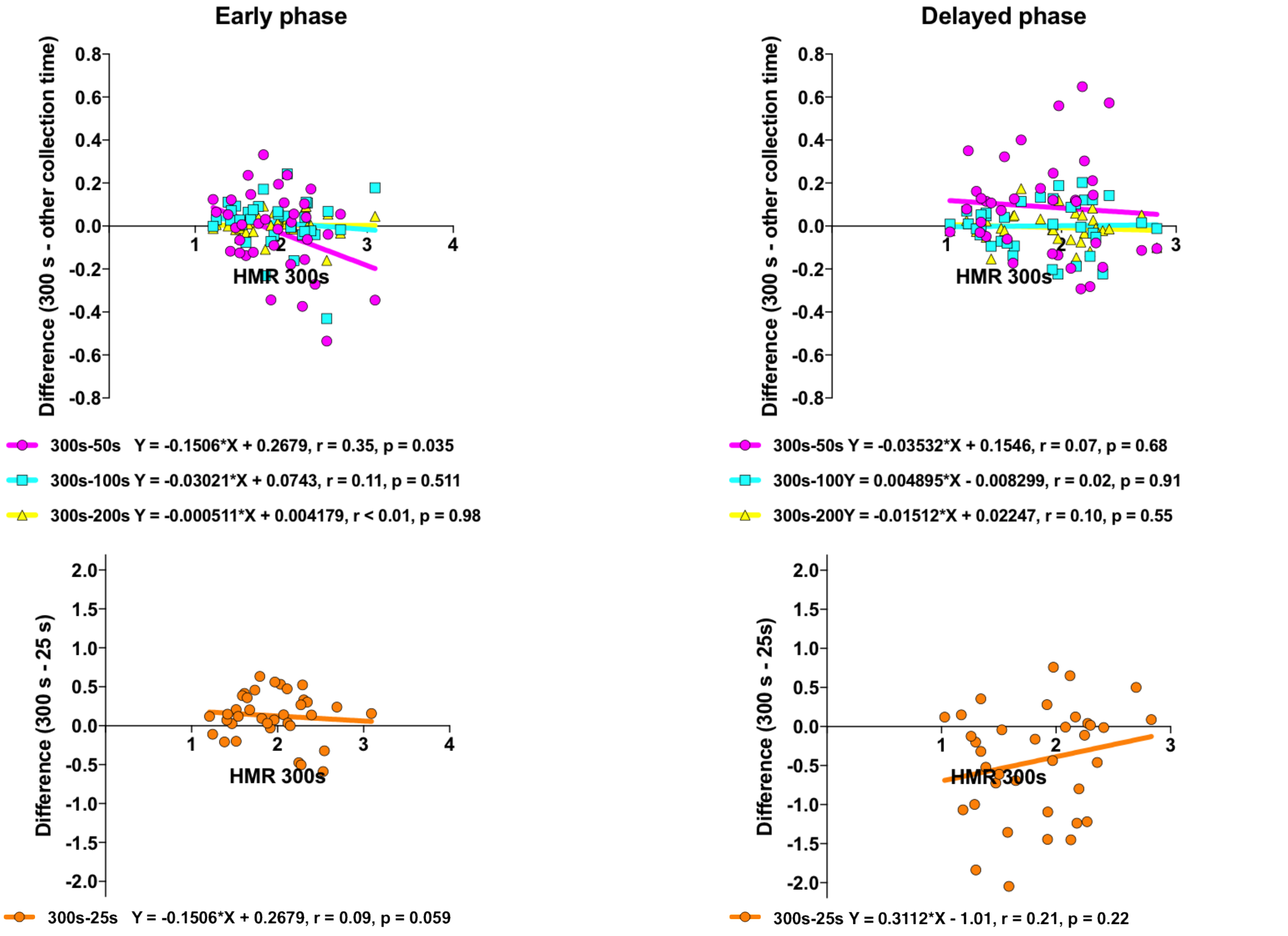

Supplement: Supplementary file 1 — Figure S1. Details of phantom models. Figure S2. Correlation between reference HMRs and difference of HMRs (300 s − other collection time) (PDF 407 kb) [file 13550_2019_491_MOESM1_ESM.pdf]
